# Supplementary figures and images for: Characterization of bacterioplankton communities and quantification of organic carbon pools off the Galapagos Archipelago under contrasting environmental conditions
Source: PeerJ. 2018 Dec 3;6:e5984. doi: 10.7717/peerj.5984 (PMC6282939; doi:10.7717/peerj.5984)

## Free-living

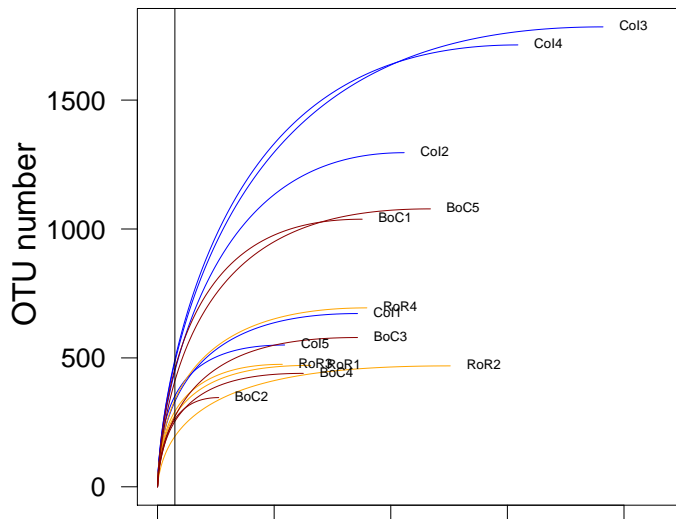

## Particle-attached

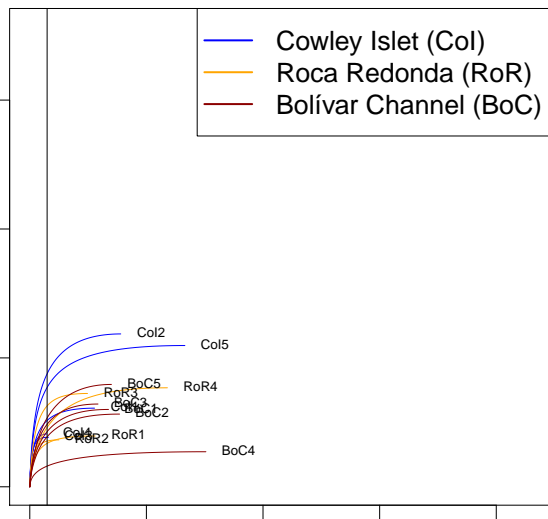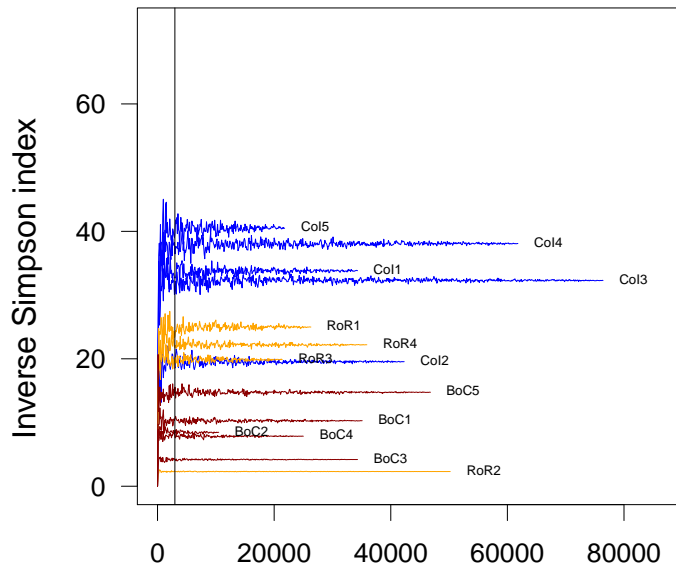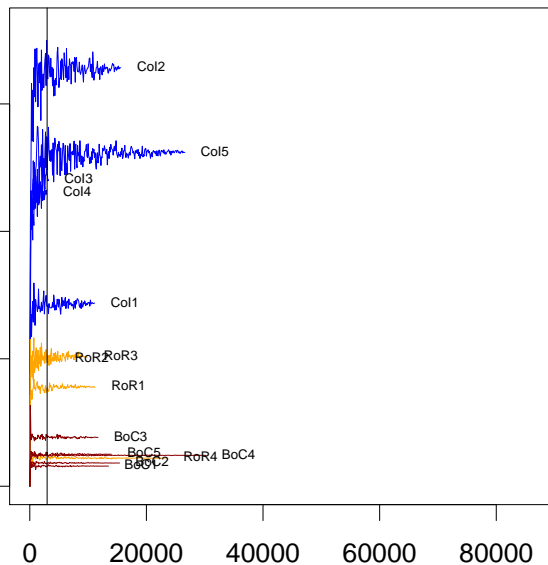

Number of sequences

Supplement: Figure S1 — Sample names specify replicate number at each of the three sampling sites: Cowley Islet (CoI1-5), Roca Redonda (RoR1-4), and Bolivar Channel (BoC1-5). The vertical line indicates the minimum library size of the data set. [file peerj-06-5984-s001.pdf]

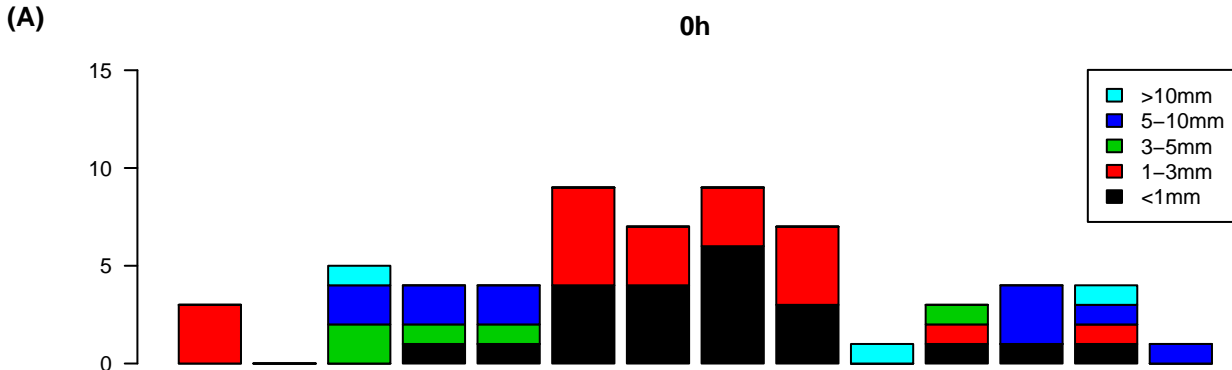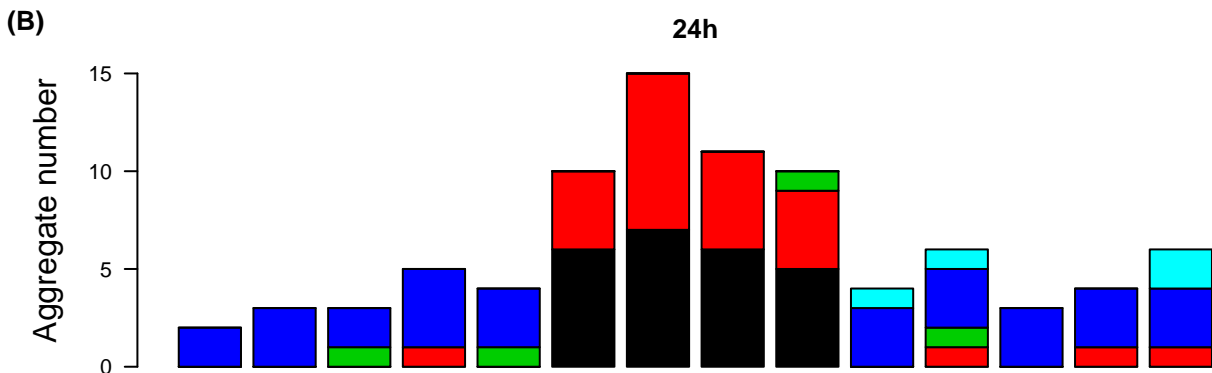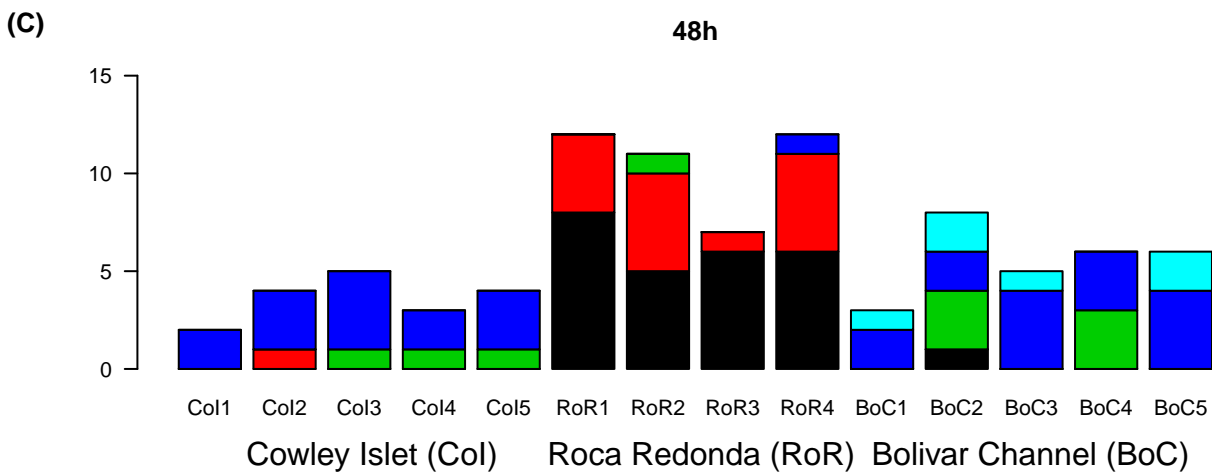

Supplement: Figure S2 — (A) Beginning of the experiment (0 h). (B) After 24h. (C) End of the experiment (48h). Aggregates were grouped into five size classes based on their approximate diameter. [file peerj-06-5984-s002.pdf]

**(A)****Free-living**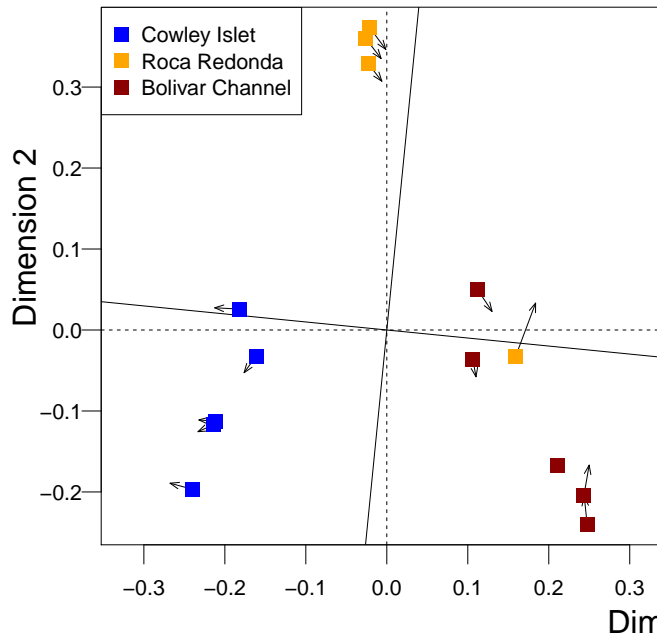**(B)****Particle-attached**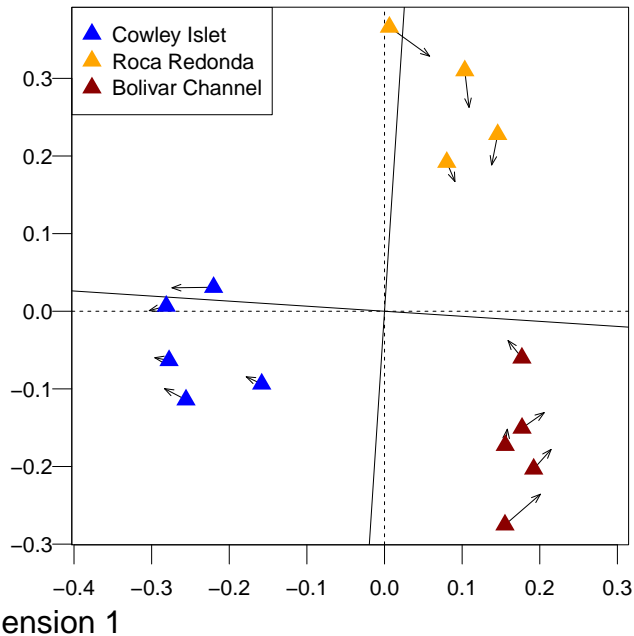

Supplement: Figure S3 — (A) Free-living bacterial communities, correlation of the symmetric Procrustes rotation: 0.99 (p = 0.001). (B) particle-attached bacterial communities, correlation of the symmetric Procrustes rotation: 0.99 (p = 0.001). Points show coordinates of the RDA ordination with sampling site as explanatory variable with arrows pointing towards the coordinates of the RDA ordination with temperature and pH as explanatory variables. [file peerj-06-5984-s003.pdf]

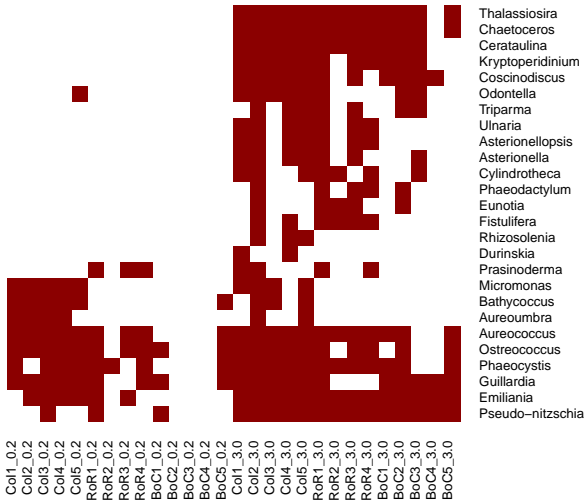

Supplement: Figure S4 — Sample names specify replicate number at each of the three sampling sites: Cowley Islet (CoI1-5), Roca Redonda (RoR1-4), and Bolivar Channel (BoC1-5), and size fraction: free-living (>0.2 µm), particle-attached (>3.0 µm). Absence (white) and presence (red) of phytoplankton genera was estimated based on best BLAST hits of 16S chloroplast sequences to 16S chloroplast sequences in the NCBI refseq database (date accessed 27.06.2017) at a sequence similarity of at least 93%. Genera of terrestrial and macroalgal origin were removed. The 16S primer set used in this study is expected to cover 80% of the chloroplast sequences in the SILVA ribosomal RNA gene database (SILVA test prime, https://www.arb-silva.de/search/testprime/, date accessed 01.10.2018). [file peerj-06-5984-s004.pdf]
